# Determinants of Evidence Implementation by Nurses: #Evidencer Model for the Use of Evidence-Based Practice (#EvidencerMUSEBP)—A Structural Equation Model

**DOI:** 10.1155/2024/7246547

**Published:** 2024-02-28

**Authors:** Maria Ruzafa-Martínez, Serafin Fernández-Salazar, César Leal-Costa, Antonio Jesús Ramos-Morcillo

**Affiliations:** ^1^University of Murcia, Department of Nursing, El Palmar 30120, Murcia, Spain; ^2^Andalusia Care Strategy, Andalusian Health Service, AGS Northeast Jaén, Úbeda, Spain

## Abstract

Existing studies have identified specific factors influencing some dimensions of evidence-based practice (EBP) competence and use. However, the way these factors interact still needs to be clarified. The purpose of the study was to test a model based on the Determinant Frameworks that explain the relationships and the direct pathways between the characteristics of the nurses, the context, and the implementation strategies and the dimensions of EBP competence, attitude, knowledge, skills, and use of EBP. A cross-sectional study was carried out in Spain during January and February 2020, involving 2,370 nurses employed in public health centers across all autonomous communities within the National Health System. An online survey was administered to gather data, addressing various topics related to the nurses' characteristics, the context in which they worked, the implementation strategy, and their competence in evidence-based practice (EBP). As depicted in the conceptual framework, a structural equation model was constructed to test the hypothesized relationships among key study variables. The model obtained showed a good fit (*χ*^2^/*df* = 3.20, *p* < 0.001; RMSEA = 0.030 [90% CI 0.025, 0.036]; CFI = 0.989; GFI = 0.990; TLI = 0.983). The context, more specifically, the dimensions of nurse participation in the center's affairs, nursing foundations for quality of care, nurse manager ability leadership and support of nurses, and implementation strategy have a direct and positive effect on EBP use. Training in EBP, reading scientific articles, and having a doctorate are associated with higher competence and knowledge in EBP. The final fit shows the #Evidencer model for the use of EBP (#EvidencerMUSEBP) with two main components: the contextual and strategic factors that influence the implementation of EBP and the characteristics of the professionals, such as their training and reading of articles, which have an impact on EBP competence. This model could guide healthcare organizations in proposing comprehensive interventions to improve EBP use and the competency of nurses.

## 1. Introduction

The competency of health professionals in evidence-based practice (EBP) plays a fundamental role in adopting and implementing EBP in clinical settings [1]. Recent research has shown, in general terms, a lack of EBP competency in nurses in many countries and practice settings [2–4]. EBP competency comprises four dimensions: attitudes, knowledge, skills, and use of EBP, which can reach different levels of progress in professionals [5]. The literature on the subject shows that health professionals, including nurses, have positive attitudes and beliefs about the importance and value of EBP for improving the care of patients and moderate levels of their EBP knowledge and skills. However, an adequate level in these dimensions does not necessarily result in changes in behavior, as the use of EBP in daily practice is generally low in all disciplines [6].

Nurses' EBP competency is intricately linked to individual characteristics, the work environment, and implementation strategies. Examining this relationship provides a more comprehensive understanding of how various factors influence the adoption and application of EBP.

First, professional characteristics, such as age and educational level, are positively associated with EBP competence. Research suggests that younger nurses and those with higher education levels exhibit stronger competence in EBP [3, 7]. More specifically, a Master's degree is associated with enhanced EBP knowledge and use [8]. In addition, specific EBP training has been associated with positive beliefs about EBP [8], and having experience in research has been associated with EBP knowledge and skills [9]. The clinical competency and professional values of nurses, as well as their role as mentors for nursing students, are key drivers of competence in this area [10, 11].

Second, the work context plays a crucial role. Organizational factors, such as the availability of resources and institutional support, have been shown to influence EBP competence [9]. Specifically, active participation in the center's affairs and leadership roles has significantly influenced EBP competence [12]. Furthermore, access to resources such as the Internet [13] and bibliographical databases [8] contributes to competence in this area. There are factors such as working in a magnet hospital that show contrary results depending on the country. A study conducted in Saudi Arabia led to an association with attitude towards EBP [14]. In contrast, a study conducted in the USA showed a lack of differences in the competencies of nurses, regardless of whether they worked in a magnet hospital or not [3].

Third, implementation strategies play a crucial role in EBP competence. Specific EBP training and the presence of specialized mentors have been associated with positive beliefs and increased knowledge of EBP [8]. Moreover, research has demonstrated that mentorship and an organizational culture supportive of EBP positively impact professionals' competence in EBP [10, 11].

As highlighted, research should test models to determine which variables have the most influence on EBP [3]. Existing studies have identified specific factors that could influence some of the dimensions of EBP competence. However, the underlying mechanisms of these relationships and how these factors interact between them still need to be clarified. Up to the present, two studies have been conducted with nursing professionals who tried to develop an explanatory model about the factors associated with competence and implementation of EBP. The first of these was conducted in the USA and was based on the ARCC© Model. The results showed that EBP culture and mentorships were key variables that directly affected the knowledge, beliefs, competence, implementation, work satisfaction, and retention of nurses [15]. On the other hand, the second study, conducted in Saudi Arabia, used a conceptual framework developed from published background works [16]. The skills and beliefs about EBP were the main factors related to their use and were also mediated by factors such as the EBP training of the nurses. The facilitators and barriers also had a significant impact on the application of the EBP [16]. However, these results have a limited generalization to other cultural settings. Cultural factors influence EBP adoption in healthcare professionals by shaping attitudes toward authority, communication styles, beliefs about health, and the emphasis on collectivism or individualism. Addressing cultural nuances is crucial for tailoring effective implementation strategies. Besides, there are variables that were not found in either model that could be interesting to consider.

Among the theories and conceptual frameworks developed to explain factors that influence the implementation of EBP, the Determinant Frameworks [17] present elements that are adequate for the establishment of an initial conceptual framework that allows testing the influence of certain factors on the competency and use of EBP by nurses. In general terms, these frameworks include five types of determinants: implementation object, characteristics of the professionals, end users, context, and strategy for facilitating the implementation and recognize, based on a systemic approach, the existence of relationships within and between the different levels, although the relationships between these determinants still need to be clarified [18].

**Figure 1:**
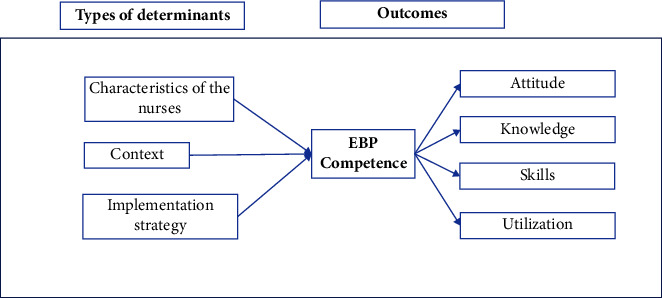
Conceptual model. The relationship between types of determinants and EBP competence.

In 2020, we conducted a national study in Spain with nurses, in which many variables that monitored three of the determinants mentioned in that model were measured. Specifically, these were the characteristics of the professionals, the context, and the strategy for facilitating the implementation [4]. Beginning with the initial conceptual framework proposed [17], the starting hypothesis to be tested was the existence of a positive relationship between the characteristics of the professionals, the context, and the strategy for facilitating the implementation with EBP competence and, at the same time, with the dimensions that shape it (Figure 1). The great heterogeneity between the studies investigating the factors and determinants of the competence and use of EBP does not allow us to be more specific a priori. Therefore, the purpose of the present study was to develop and test a model supported by the Determinant Frameworks that could explain the relationships and the direct pathways between the nurses' characteristics, the context, and the strategies of implementation, with the dimensions of the EBP competence, attitude, knowledge, skills, and use of EBP.

## 2. Materials and Methods

### 2.1. Design

The EBP competence of nurses was evaluated with the data from an observational, cross-sectional, and national study conducted in Spain between January and February 2020 [4]. This timeframe offers a unique insight into the EBP competence of nurses in a prepandemic context. At the same time, this study design allows for the simultaneous collection of data at a single point in time, offering a snapshot of the relationships and variables of interest in a same country.

### 2.2. Participants and Setting

The study included nurses who worked at public health centers from the National Health System in all the autonomous communities in Spain. The following were the selection criteria: nurses currently employed at public health centers affiliated with the NHS, with at least one year of work experience, and working either at a hospital or primary care center with any type of contract.

Data were collected through an online survey using a collaborative national campaign named #Evidencer. The sampling was nonprobabilistic, with voluntary participation among professionals who chose to engage after receiving the invitation. The campaign extended invitations to nurses nationwide via social media, professional associations, trade unions, and scientific organizations to enhance representation.

### 2.3. Variables and Instruments

The online survey included questions about the characteristics of the nurses, context, strategy for facilitating the implementation, and EBP competence.

### 2.4. Characteristics of the Nurses

The sociodemographic and professional variables of the nurses included were age, sex, time since completing the Nursing degree, professional experience, level of education that includes bachelor, specialist nurse (refers to formal and officially recognized training that equips professionals with specific clinical competencies in various areas such as obstetrics, community health, and pediatrics), master's degree, and doctorate degree, training on EBP, number of articles read in the last month, nursing students' mentor, and use of the Internet and other digital tools to access scientific information.

### 2.5. Context

To analyze the organizational context of clinical practice, the Spain-validated version of the questionnaire Practice Environment Scale of the Nursing Work Index (PES-NWI) was utilized to measure the context of nursing practice in health organizations. This instrument has been validated in Spanish in the hospital and primary care contexts [19, 20]. Both versions are similar with the same number of items and the original five-factor model. The Spanish versions of the questionnaire demonstrate robust psychometric properties, including validity and reliability. As outlined in these articles, the validation process underscores the instrument's capacity to effectively measure the nursing work environment in the Spanish context of community and hospital. In order to ensure accuracy, we used a neutral version of the items or employed two terms where necessary, to accommodate nurses from both contexts. The questionnaire contained 31 items organized into five factors: factor I includes nurse participation in the center's affairs (9 items); factor 2 includes nursing foundations of quality of care (10 items); factor 3 includes nurse manager ability, leadership, and support (5 items); factor 4 includes staffing and resource adequacy (4 items); and factor 5 includes nurse-physician relations (3 items). The items were scored with a Likert scale with four response options (from “strongly disagree” to “strongly agree”).

In addition to the PES-NWI, we looked at other factors related to the work environment, such as employment status, type of contract, work location, context of care (hospital or primary care), and access to the Internet while at work.

### 2.6. Strategy for Facilitating the Implementation

This determinant was evaluated by asking the nurses if they worked at a center that was part of the “Best Practice Spotlight Organization (BPSO®) implementation program.” These are healthcare centers that participate in the international Registered Nurse' Association of Ontario (RNAO) program for the implementation of Clinical Practice Guidelines (CPGs). This program has been implemented in Spain since 2012, and centers are selected through a competitive process; the centers present the proposals for implementing and evaluating the RNAO CPG in 3 years. The implementation methodology followed in all centers is an adaptation of the knowledge to action model, which includes the following phases: (a) identify the problem and select the available knowledge; in this case, those provided by the CPG; (b) adapt the recommendations to the local setting; (c) assess the obstacles and the facilitators of the use of knowledge; (d) plan and execute the application; (e) supervise the use of knowledge; (f) evaluate the results to determine the success of the application; and (g) develop sustainability strategies [21].

### 2.7. EBP Competence

To assess the EBP competence, the “Evidence-Based Practice Competency Questionnaire, Professional version (EBP-COQ-Prof©)” was utilized. This tool was validated in Spanish, with adequate validity and reliability. It allows measuring the self-perceived EBP competence of nurses [5]. Cronbach's *α* for each scale dimension was 0.888, indicating good internal consistency. A final model was tested with four oblique factors and 35 items. The model fit indices were *χ*^2^ = 1,935.92 (*df* = 554; *p* < 0.001), *χ*^2^/*df* = 3.49, CFI = 0.932, TLI = 0.927, and RMSEA = 0.093 (90% CI = 0.097–0.108). Factors I is attitude (8 items, range 8–40); factor II is knowledge (11 items, range 11–55); factor III is skills (6 items, range 6–30); and factor IV is utilization (10 items, range 10–50). The items are scored using a Likert scale from 1 to 5 (from “strongly disagree” to “strongly agree”). The overall score for evidence-based practice (EBP) competence ranges from 35 to 175 points, with a higher score indicating greater competence.

### 2.8. Analysis of Data

Data analysis was performed using the SPSS statistical package version 22.0 and AMOS version 20 (IBM Inc., 2013, NYC). Descriptive statistics were calculated to describe the participants' background characteristics (e.g., basic demographic variables and work-related variables) and key study variables (i.e., EBP competence, context, and strategies for facilitating the implementation of PBE programs). We further examined if any background characteristics were associated with key study variables using one-way ANOVA (for the EBP competence). Pearson's correlation coefficients were also calculated to examine the associations between key study variables.

A structural equation model (SEM) was constructed to test the hypothesized relationships among key study variables as depicted in the conceptual framework (Figure 1). The variables showed adequate normality for the maximum likelihood estimation (MLE) method, i.e., skewness >2-3 and kurtosis >7–10 [22]. The significance of the regression coefficients was evaluated after estimating the parameters. The effects with *p* ≤ 0.05 were considered significant. The fit of the model was evaluated using *χ*^2^/*df* <5, the root mean square error of approximation (RMSEA) values ≤0.08, and the comparative fit (CFI), goodness of fit (GFI), and Tucker–Lewis index (TLI) values ≥0.90 indicate a good fit [23].

### 2.9. Ethical Considerations

The study was approved by the Ethics Committee of the University of Murcia (ID: 2540/2019). The nurses were invited to participate voluntarily through an online survey. They were informed about the study's objectives, making it clear that their participation was completely anonymous and that they provided their consent to participate by sending it.

## 3. Results

The nurses who completed the survey (*n* = 2370) had a mean age of 41.3 (SD = 9.8), a high percentage were women (79.80%), slightly more than half had a Master's degree (55.6%), and about 30% worked in an organization that was implementing the BPSO® program. The remaining sociodemographic variables are shown in Table 1.

With respect to the bivariate results, the categorical variables that were observed to have a statistically significant relationship with the dimensions from EBP competence are shown in Table 2. Relationships were observed between almost all dimensions from the EBP-COQ Prof © questionnaire with level of education, EBP training, reading scientific articles, and being a nursing student mentor. Also, sex, use of social networks, having internet access at work, and working in a BPSO® center were associated with the utilization dimension and total competency.

Significant correlations were also observed between the quantitative sociodemographic variables, the dimensions from the PES-NWI, and the dimensions from the EBP-COQ Prof© questionnaire (Table 3). Age, the time since completing the nursing degree, and work experience showed significant and inverse bivariate correlations with attitude, knowledge, total EBP competence, and the dimension from the PES-NWI collegial nurse/physician relation, while positive correlations were obtained with nurse participation in the center's affairs and nursing foundations for quality of care. The dimensions from the PES-NWI showed correlations with the dimensions from the EBP-COQ that oscillated between 0.020 and 0.094 with attitude, between 0.055 and 0.151 with knowledge, and between 0.134 and 0.217 with skills. The dimension use of EBP showed the strongest correlations, with all the dimensions from the PES-NWI obtaining values between 0.306 and 0.535.

### 3.1. Structural Equation Modeling

#### 3.1.1. Testing the Initial Hypothesized Model

**Figure 2:**
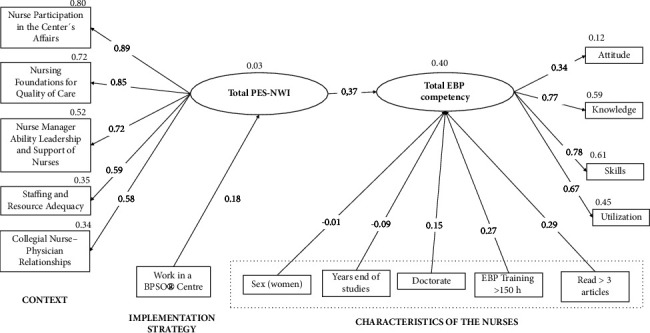
Initial model with standardized parameter estimates.

The preliminarily hypothesized model (Figure 2) showed a poor fit (*χ*^2^/df = 16.62, *p* < 0.001; RMSEA = 0.081 (IC del 90% 0.077, 0.085); CFI = 0.885; GFI = 0.938; TLI = 0.837). After evaluating modification indices and parameter estimates, numerous paths were nonsignificant; subsequently, they were removed to make the measurement model more theoretically parsimonious.

#### 3.1.2. Testing the Modified Model

**Figure 3:**
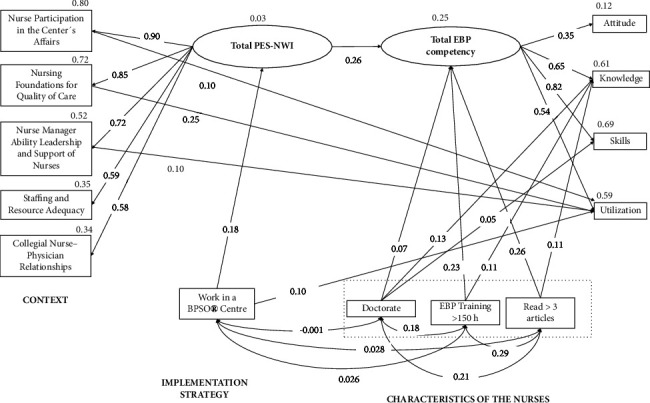
Modified model with standardized parameter estimates: #EvidencerMUSEBP model.

The influencing factors on EBP competence were specified (Figure 3 and Table 4). The modified model showed a good fit (*χ*^2^/*df* = 3.20, *p* < 0.001; RMSEA = 0.030 (90% CI 0.025, 0.036); CFI = 0.989; GFI = 0.990; TLI = 0.983). Explicitly, EBP competence was significantly influenced by work context (*β* = 0.26, *p* < 0.001), level of education (Doctorate) (*β* = 0.07, *p* < 0.001), EBP training >150 hours (*β* = 0.23, *p* < 0.001), and read >3 articles (*β* = 0.26, *p* < 0.001). The study findings show that work in a BPSO® center had an indirect effect on EBP competence. In total, the factors explained 25% of the variance on EBP competence.

The knowledge dimension of EBP competence was significantly influenced by level of education (Doctorate) (*β* = 0.13, *p* < 0.001), EBP training >150 hours (*β* = 0.11, *p* < 0.001), and read >3 articles (*β* = 0.11, *p* < 0.001). In addition, the abovementioned variables, work in a BPSO® center and the work context, had an indirect effect on the knowledge dimension of EBP competence. In total, the factors explained 61% of the variance on knowledge dimension.

Finally, the utilization dimension of EBP competence was significantly influenced by Nurse Participation in the center's affairs (*β* = 0.10, *p* < 0.001), Nursing Foundations for Quality of Care (*β* = 0.26, *p* < 0.001), Nurse Manager Ability Leadership and Support of Nurses (*β* = 0.10, *p* < 0.001), and work in a PBSO® Center (*β* = 0.10, *p* < 0.001). Furthermore, work in a BPSO® center, work context, level of education (Doctorate), EBP training >150 hours, and reading> 3 articles had an indirect effect on the utilization dimension of EBP competence. The factors explained 59% of the variance in the knowledge dimension (Table 4).

## 4. Discussion

Following the Determinant Framework [17], our study presents the first empirical model that tested the relationship of certain variables associated with the characteristics of the professionals, the context, and the implementation strategies with EBP competence and utilization of a national sample of nurses in Spain whose sociodemographic and professional characteristics aligned with those of Spanish nurses employed in public health centers [24]. The final fit shows a model mainly linked to the utilization of EBP by clinical nurses. This model will be referred to as the #Evidencer model for the use of EBP (#EvidencerMUSEBP).

The #EvidencerMUSEBP model consists of two main components. The first component is related to the utilization of evidence-based practice (EBP), which includes determinants associated with the context and implementation strategy. The second component is related to the characteristics of professionals, such as their training and reading of articles. This component is directly linked to EBP competence, knowledge, and skills. The #EvidencerMUSEBP model suggests that although professionals may possess sufficient knowledge and skills in EBP, it may translate into something other than an equivalent use of EBP. This is consistent with findings from many studies [6]. It can also explain why interventions that solely focus on training professionals only improve their knowledge and skills without significantly impacting the use of EBP [25, 26]. Without a doubt, the model is complex and requires a systematic strategy that can synchronously and cohesively influence different factors to improve the use of EBP. This conclusion aligns with the findings of a recent review on the implementation of change in nursing practice [27].

In our study, we found that the determinants “context” and “implementation strategy” accounted for 59% of the variation in the utilization of evidence-based practice (EBP). This indicates a considerable effect size. We use the Practice Environment Scale of the Nursing Work Index (PES-NWI) to evaluate the clinical environment, a reliable tool widely used to assess nursing practice environments across multiple countries [28]. Also, this instrument includes the most common dimensions of the context described in the determinant frameworks widely used in evidence science; the majority of the frameworks outlined contextual determinants that could be ascribed to organizational support, financial resources, and social relations and support, as well as leadership, organizational culture, and climate [29]. Our findings showed that the overall score in the practice environment was directly related to EBP competence, which aligns with previous studies [12]. In addition, we discovered that the dimensions of nurse participation in the center's affairs, nursing foundations for quality of care, and nurse manager ability leadership and support of nurses have a positive, direct effect, more specifically on the utilization of EBP. This means that promoting the participation of nurses in the institution's internal governing body, political and committee decisions, providing them with promotion opportunities, having good nursing managers and leaders, and institutions having a nursing philosophy directly influence the use of EBP in clinical practice. Our findings are consistent with other recent studies [30–33].

Regarding the dimensions evaluated in the context, it is surprising that the dimension of staffing and resource adequacy, commonly viewed as a barrier against the use of research in clinical practice [34, 35], did not influence the use of EBP. This finding is consistent with previous studies [36]. Experts have pointed out that resource and personnel availability may be favorable for applying EBP, but they must be accompanied by leadership, promotion opportunities, and participation in the institution for EBP use to be effective [29]. This idea emphasizes the fact that we are dealing with a complex model, and a systemic strategy that can influence the different factors in a synchronous and coordinated manner is needed to address it.

Concerning the determinant of “strategy for facilitating implementation,” which is defined as the methods or techniques utilized to improve the adoption, application, and sustainability of a program or clinical practice [37], it has been assessed through the implementation program of clinical practice guidelines named BPSO® of the RNAO. The model showed a positive and direct relation between participation in this program and the use of EBP. This strategy involves the institution in both the implementation and development of Clinical Practice Guidelines (CPGs). It requires the support of executive directors and nurse managers at healthcare centers, promoting teamwork and a culture of change. These aspects are deemed fundamental for successfully implementing evidence-based practices [38]. In line with our results, applying the BPSO® program has shown favorable results in using EBP in clinical practice in health centers in Spain [39, 40] and other countries [41, 42]. These findings, consistent with previous studies, provide new empirical evidence supporting the link between organizational support for innovation and the adoption of innovative practices [43].

The #EvidencerMUSEBP model also suggests that the implementation strategy indirectly affects EBP competence and knowledge, mediated by the practice environment, suggesting that the strategy to facilitate the implementation of evidence also positively influences these two aspects at a secondary level. Empirical evidence has demonstrated that training professionals on aspects related to the culture of change and EBP knowledge included in the BPSO® program leads to improvements in EBP competence and knowledge [44]. These results confirm that the successful application of the EBP strategy tends to require a process of active change directed towards the use of the intervention by individuals and the organization [45] to achieve a change in the practice environment that, at the same time, influences the competence of professionals.

Concerning the determinant characteristics of the nursing professionals related to the training and direct contact with scientific updating and the overall score of the PES-NWI (context), the determinants showed a direct relationship with EBP competence, explaining 25% of the variance. In addition, the variables related to the characteristics of the professional (having a doctorate, having more than 150 hours of EBP training, and reading more than three articles per month), together with the overall score of the PES-NWI mediated by its effect on the general EBP competence, explained 61% of the variance, while having a doctorate also influenced EBP skills, although the relationship was weak. These findings are significant, as negative feelings or a lack of interest in research by nursing professionals have been described [46], so activities that promote the association between research and nursing practice should be promoted starting at the initial levels of nurse training. Also, the final model did not retain variables associated with the sociodemographic characteristics of the professionals, such as gender, age, or years of work experience, which are significant in previous studies [47]. This omission suggests that in the fitting of the final model, these variables did not play a determinant role. Scientifically, these variables may exert limited influence on evidence-based practice (EBP) competence and utilization compared to factors more directly linked to the profession, environment, and implementation strategies. Moreover, their exclusion may contribute to a more parsimonious and specific model, mitigating issues related to multicollinearity and emphasizing determinants more pertinent to the effective adoption of EBP in clinical settings.

The results contribute towards prioritizing the determinants on which health organizations should propose interventions to improve EBP use and competence. The #EvidencerMUSEBP and the associations established between the determinants studied show that it is vital to consider the characteristics of the professionals, the context, and the implementation strategies in a manner that is integrated and nonfragmented, as the successful application of EBP depends on the combinations of different determinants. Adopting an excessively reductionist approach, in which an intervention is conducted in a single variable, will not have the ability to influence the improvement of the use of EBP. Two or more determinants can be combined to create efficient effects and with an amplified effect that acts on nurses' use, knowledge, skills, and EBP competence.

The study's findings have notable implications for nurse managers, emphasizing the need for leadership development to promote evidence-based practices. Nurse managers can play a pivotal role in shaping organizational culture, fostering participation, and strategically engaging in programs like BPSO® for successful EBP implementation. Customized implementation strategies, continuous professional development, and a focus on creating supportive environments are key considerations for nurse managers aiming to enhance EBP competence among their teams.

## 5. Limitations

It is important to acknowledge certain study limitations that can affect the interpretation of its results. First, the selection of participants relied solely on nurses' willingness, and data collection was done through online surveys. These two factors may have introduced bias in the selection process as the characteristics of the nurses who participated may differ from those who chose not to participate or those who do not have access to the internet. We could not identify whether the nurses' master's degree was professionalizing or research based. This difference could affect the number of research hours and, consequently, influence the results. Second, conducting a more detailed examination of the potential limitations associated with the positive correlation between participation in the BPSO® program and the use of Evidence-Based Practice (EBP) would be helpful. Future research should focus on exploring contextual factors that may influence the effectiveness of the program, such as differences in organizational structure, nursing contexts like hospital and primary care, varying levels of engagement among participants, or potential challenges in implementing the program. This deeper analysis will provide a more balanced perspective and facilitate a better understanding of the program's real-world applicability and potential areas for improvement.

Furthermore, while the study evaluated multiple variables related to the organizational context, it is essential to note that these variables were based on the perceptions of the participants as proposed by the questionnaire utilized, and this may not have comprehensively captured all the relevant aspects of the work environment that could have influenced EBP competence. For future research, it is recommended to test the #EvidencerMUSEBP separately in hospital and primary care contexts and compare results across different regions. In addition, including additional determinants of the model that were not analyzed in this study, such as the type of evidence and the end users, and to evaluate the impact of these factors in the #EvidencerMUSEBP would provide further insights.

## 6. Conclusions

The #EvidencerMUSEBP model incorporates characteristics of professionals, context, and implementation strategies, demonstrating a solid fit. This model provides empirical evidence that directly associates the characteristics of the nursing professionals, such as a high level of education, reading articles, and EBP training, with EBP knowledge and skills, thereby indirectly impacting the use of evidence.

On the other hand, the context conceived as the practice environment, which includes a nursing perspective, and is backed by institutional leaders and organizations that promote the feeling of belonging of the professionals, together with strategies such as the implementation of the CPG BPSO® program, exerts a direct influence on EBP adoption. These factors, at the same time, exert an indirect effect on EBP competence and knowledge.

The study emphasizes the vital role of leadership for nurse managers in promoting evidence-based practices, highlighting the need for customized strategies and continuous professional development to enhance competence within healthcare teams. A key aspect is that healthcare services managers and providers must internalize the need to jointly address these elements, recognizing that improvement in EBP requires comprehensive, synchronous, and coordinated actions on all fronts.

## Figures and Tables

**Table 1:** Sociodemographic and professional variables of the sample (*N* = 2370).

	*M*	SD
Age (years)	41.3	9.8
Time since completing the nursing degree (years)	19.4	10.0
Professional experience (years)	17.6	10.1

	*n*	%

Sex		
Male	478	20.2
Female	1892	79.8
Educational level		
Bachelor	945	39.9
Master	1004	42.4
Clinical nurse specialist	245	10.3
Doctoral	176	7.4
Employment status		
Eventual	529	22.3
Interim	562	23.7
Permanent	1279	54.0
Type of contract		
Full time	2141	90.3
Part-time	229	9.7
Work setting		
Urban (>50,000 inhabitants)	1620	68.4
Suburban (between 10,000 and 50,000 habitants)	541	22.8
Rural (<10,000 habitants)	209	8.8
Context of care		
Hospital	1660	70.0
Primary care	710	30.0
Training on EBP *n* (%)		
None	350	14.8
<40 hours	582	24.6
40–150 hours	694	29.3
>150 hours	744	31.4
Number of articles read in the last month		
0	384	16.2
1 to 3	1013	42.7
>3	973	41.1
Working at a BPSO® center		
Yes	635	26.8
No	1735	73.2
Nursing students' mentor		
Yes	1163	49.1
No	1207	50.9
Use of the Internet and other digital tools to access scientific information		
Yes	1966	83.0
No	404	17.0
Access to the Internet at work		
Yes	2144	90.5
No	226	9.5
Place where accessing the Internet most frequently to consult information		
Home	1855	78.3
Work	515	21.7

M: mean; SD: standard deviation.

**Table 2:** Comparison of the sociodemographic and professional variables with the dimensions and total EBP-COQ-Prof©.

	*N*	EBP attitude			EBP knowledge			EBP skills			EBP utilization			EBP total		
		M	SD	*p*value	M	SD	*p*value	M	SD	*p*value	M	SD	*p*value	M	SD	*p*value
Male	478	4.65	0.39	0.154	3.68	0.80	<0.001	3.99	0.56	<0.001	3.31	0.64	0.619	3.85	0.48	<0.001
Female	1892	4.68	0.37		3.46	0.81		3.86	0.64		3.29	0.63		3.76	0.48	

*Educational level*																
(1) Bachelor	945	4.64^2-3-4^	0.39	0.004	3.77^2-3-4^	0.78	<0.001	3.75^2-3-4^	0.55	<0.001	3.22^2-4^	0.60	<0.001	3.62^2-3-4^	0.46	<0.001
(2) Master	1004	4.69^1^	0.36		3.62^1-3-4^	0.76		3.93^1-4^	0.57		3.32^2-3-4^	0.65		3.83^1-4^	0.46	
(3) SN	245	4.69^1^	0.39		3.77^1-2-4^	0.66		3.97^1-4^	0.49		3.34^1^	0.61		3.89^1-4^	0.42	
(4) PhD	176	4.73^1^	0.38		4.30^1-2-3^	0.55		4.25^1-2-3^	0.48		3.45^1^	0.65		4.15^1-2-3^	0.40	

*Training on EBP*																
(1) None	350	4.58^2-3-4^	0.47	<0.001	2.86^2-3-4^	0.76	<0.001	3.63^2-3-4^	0.59	<0.001	3.00^2-3-4^	0.60	<0.001	3.43^2-3-4^	0.45	<0.001
(2) <40 hours	582	4.66^1-4^	0.37		3.21^1-3-4^	0.73		3.75^1-3-4^	0.53		3.19^1-3-4^	0.60		3.63^1-3-4^	0.45	
(3) 40–150 hours	694	4.68^1-4^	0.37		3.58^1-2-4^	0.69		3.88^1-2-4^	0.54		3.34^1-2-4^	0.61		3.81^1-2-4^	0.43	
(4) >150 hours	744	4.73^2-3-4^	0.32		3.96^1-2-3^	0.70		4.12^1-2-3^	0.52		3.47^1-2-3^	0.62		4.02^2-3-4^	0.42	

*Number of articles read in the last month*																
(1) 0	384	4.49^2-3^	0.54	<0.001	2.81^2-3^	0.78	<0.001	3.47^2-3^	0.60	<0.001	2.94^2-3^	0.61	<0.001	3.35^2-3^	0.46	<0.001
(2) 1 to 3	1013	4.68^1-3^	0.32		3.39^1-3^	0.71		3.85^1-3^	0.51		3.27^1-3^	0.59		3.73^1-3^	0.41	
(3) >3	973	4.74^1-2^	0.32		3.89^1-2^	0.70		4.08^1-2^	0.51		3.45^1-2^	0.63		3.99^1-2^	0.42	

*Use of digital tools to access scientific information*																
Yes	1966	4.65	0.38	0.215	3.53	0.79	0.003	3.90	0.56	0.005	3.30	0.62	0.041	3.79	0.46	0.002
No	404	4.68	0.37		3.39	0.91		3.81	0.59		3.23	0.68		3.71	0.53	

*Access to the Internet at work*																
Yes	2144	4.67	0.38	0.820	3.51	0.81	0.458	3.89	0.57	0.600	3.31	0.64	0.002	3.78	0.48	0.099
No	226	4.68	0.32		3.46	0.79		3.87	0.50		3.17	0.58		3.73	0.43	

*Nursing student mentor*																
Yes	1163	4.68	0.39	0.385	3.58	0.82	<0.001	3.97	0.56	<0.001	3.36	0.65	<0.001	3.83	0.49	<0.001
No	1207	4.67	0.37		3.43	0.80		3.81	0.56		3.23	0.61		3.72	0.46	

*Working in a BPSO® center*																
Yes	635	4.68	0.36	0.833	3.55	0.83	0.102	3.94	0.54	0.008	3.51	0.60	<0.001	3.86	0.45	<0.001
No	1735	4.67	0.38		3.49	0.77		3.87	0.57		3.21	0.63		3.74	0.48	

SN: specialist nurse; M: mean; SD: standard deviation; ^1,2,3,4,5^the category of nurses with which it has statistically significant differences (*p* < 0.000) in the pairwise analysis of the Games–Howell post hoc comparison test.

**Table 3:** Relationships among sociodemographic variables, PES-NWI dimensions, and EBP-COQ Prof © dimensions.

		Years end of studies	Professional experience	Nurse participation in center's affairs	Nursing foundations for quality of care	Nurse manager ability. Leadership and nurse support	Staffing and resources adequacy	Collegial nurse/physician relationship	PES-NWI total	EBP attitude	EBP knowledge	EBP skills	EBP utilization	EBP competence
Age	*r*	0.924	0.921	0.111	0.068	0.031	0.037	−0.048	0.070	−0.077	−0.093	−0.032	0.039	−0.055
	*p* value	<0.001	<0.001	<0.001	0.001	0.134	0.074	0.020	0.001	<0.001	<0.001	0.118	0.058	0.008

Years end of studies	*r*	1	0.974	0.110	0.074	0.024	0.042	−0.051	0.070	−0.079	−0.086	−0.026	0.042	−0.049
	*p* value		<0.001	<0.001	<0.001	0.249	0.043	0.014	0.001	<0.001	<0.001	0.204	0.042	0.017

Professional experience	*r*		1	0.115	0.076	0.021	0.047	−0.046	0.073	−0.077	−0.070	−0.007	0.048	−0.035
	*p* value			<0.001	<0.001	0.309	0.021	0.024	<0.001	<0.001	0.001	0.742	0.020	0.093

Nurse participation in center's affairs	*r*			1	0.763	0.654	0.537	0.501	0.914	0.094	0.151	0.200	0.503	0.327
	*p* value				<0.001	<0.001	<0.001	<0.001	<0.001	<0.001	<0.001	<0.001	<0.001	<0.001

Nursing foundations for quality of care	*r*				1	0.609	0.490	0.499	0.896	0.083	0.133	0.210	0.535	0.330
	*p* value					<0.001	<0.001	<0.001	<0.001	<0.001	<0.001	<0.001	<0.001	<0.001

Nurse manager ability. leadership and nurse support	*r*					1	0.413	0.425	0.791	0.075	0.062	0.134	0.417	0.231
	*p* value						<0.001	<0.001	<0.001	<0.001	0.003	<0.001	<0.001	<0.001

Staffing and resource adequacy	*r*						1	0.418	0.667	0.066	0.111	0.157	0.306	0.218
	*p* value							<0.001	<0.001	0.001	<0.001	<0.001	<0.001	<0.001

Collegial nurse/physician relationship	*r*							1	0.637	0.048	0.055	0.145	0.307	0.183
	*p* value								<0.001	0.020	0.007	<0.001	<0.001	<0.001

PES-NWI total	*r*								1	0.096	0.138	0.217	0.546	0.340
	*p* value									<0.001	<0.001	<0.001	<0.001	<0.001

EBP attitude	*r*									1	0.243	0.294	0.246	0.461
	*p* value										<0.001	<0.001	<0.001	<0.001

EBP knowledge	*r*										1	0.631	0.461	0.876
	*p* value											<0.001	<0.001	<0.001

EBP skills	*r*											1	0.529	0.790
	*p* value												<0.001	<0.001

EBP utilization	*r*												1	0.773
	*p* value													<0.001

**Table 4:** Summary of the total, direct, and indirect effects of variables in the model.

Outcome variables	Independent variables	*β*	Standardized effects			Squared multiple correlations
			Direct effect	Indirect effect	Total effect	
Total EBP competence	Work context (total PES-NWI)	0.667	0.26^*∗∗*^	0	0.26^*∗∗*^	0.25
	Education level (doctorate)	0.282	0.07^*∗*^	0	0.07^*∗*^	
	EBP training >150 h	0.541	0.23^*∗∗*^	0	0.23^*∗∗*^	
	Read >3 articles	0.570	0.26^*∗∗*^	0	0.26^*∗∗*^	
	Work in a PBSO® center	0.111	0	0.046	0.046	

Knowledge dimension of EBP competence	Education level (doctorate)	6.089	0.13^*∗∗*^	0.045	0.178^*∗∗*^	0.61
	EBP training >150 h	5.087	0.11^*∗∗*^	0.152	0.264^*∗∗*^	
	Read >3 articles	5.090	0.11^*∗∗*^	0.170	0.279^*∗∗*^	
	Work in a PBSO® center	0.603	0	0.030	0.030	
	Work context (total PES-NWI)	3.629	0	0.168	0.168^*∗∗*^	

Utilization dimension of EBP competence	Work in a PBSO® center	1.470	0.102^*∗∗*^	0.091	0.194^*∗∗*^	0.59
	PES-NWI dimension nurse participation in center's affairs	3.169	0.099^*∗∗*^	0	0.099^*∗∗*^	
	PES-NWI dimension nursing foundations for quality of care	2.673	0.253^*∗∗*^	0	0.253^*∗∗*^	
	PES-NWI dimension nurse manager ability leadership and support of nurses	0.815	0.102^*∗∗*^	0	0.102^*∗∗*^	
	Education level (doctorate)	0.893	0	0.037	0.037	
	EBP training >150 h	1.716	0	0.125	0.125	
	Read >3 articles	1.806	0	0.140	0.140^*∗∗*^	
	Work context (total PES-NWI)	7.885	0	0.516	0.516^*∗∗*^	

^*∗*^ *p* < 0.05; ^*∗∗*^*p* < 0.001.

## Data Availability

The data used to support the study are available from the corresponding author upon request.

## Supplementary Materials

Supplementary Materials Estimates, covariance matrix, and correlation matrix of the study's initial and final models.
