# Supplementary material for: Determinants of Evidence Implementation by Nurses: #Evidencer Model for the Use of Evidence-Based Practice (#EvidencerMUSEBP)—A Structural Equation Model
Source: J Nurs Manag. 2024 Feb 28;2024:7246547. doi: 10.1155/2024/7246547 (PMC11919092; doi:10.1155/2024/7246547)
Supplement: Supplementary Materials — Estimates, covariance matrix, and correlation matrix of the study's initial and final models. [file 7246547.f1.zip › Final model-Estimates - covariance matrix -correlation matrix.pdf]

Pairwise Parameter Comparisons (Default model)

Variance-covariance Matrix of Estimates (Default model)

	par_1	par_2	par_3	par_4	par_5	par_6	par_7	par_8	par_9	par_10	par_11	par_12	par_13	par_14	par_15	par_16	par_17	par_18	par_19	par_20	par_21	par_22	par_23	par_24	par_25	par_26
par_1	,002																									
par_2	,002	,002																								
par_3	,002	,001	,003																							
par_4	,001	,001	,001	,002																						
par_5	,000	,000	,000	,000	,136																					
par_6	,000	,000	,000	,000	,052	,029																				
par_7	,000	,000	,000	,000	,064	,030	,046																			
par_8	,001	,001	,001	,001	-,014	-,007	-,007	,005																		
par_9	,000	,000	,000	,000	,000	,001	-,003	-,001	,051																	
par_10	,000	,000	,000	,000	,001	,000	-,002	-,001	-,031	,058																
par_11	,000	,000	,000	,000	,000	,000	,000	,000	-,001	-,005	,043															
par_12	,000	,000	,000	,000	-,010	-,005	-,006	,001	,000	,000	,000	,004														
par_13	,000	,000	,000	,000	-,011	-,006	-,007	,001	,000	,000	,000	,000	,004													
par_14	,000	,000	,000	,000	-,019	-,009	-,001	,002	-,002	-,002	,000	,002	,003	,470												
par_15	,000	,000	,000	,000	-,007	-,006	,000	,001	-,001	-,001	,000	,001	,001	,126	,084											
par_16	,000	,000	,000	,000	-,019	,001	-,001	,001	,000	-,001	,000	-,005	,001	-,017	-,001	,104										
par_17	,000	,000	,000	,000	-,019	,003	,000	,001	,002	-,002	,000	,001	-,005	-,024	-,002	-,011	,098									
par_18	,000	,000	,000	,000	,000	,000	,000	,000	,000	,000	,000	,000	,000	,000	,000	,000	,000	,000								
par_19	,000	,000	,000	,000	,000	,000	,000	,000	,000	,000	,000	,000	,000	,000	,000	,000	,000	,000	,000							
par_20	,000	,000	,000	,000	,000	,000	,000	,000	,000	,000	,000	,000	,000	,000	,000	,000	,000	,000	,000	,000						
par_21	,000	,000	,000	,000	,000	,000	,000	,000	,000	,000	,000	,000	,000	,000	,000	,000	,000	,000	,000	,000	,000					
par_22	,000	,000	,000	,000	,000	,000	,000	,000	,000	,000	,000	,000	,000	,000	,000	,000	,000	,000	,000	,000	,000	,000				
par_23	,000	,000	,000	,000	,000	,000	,000	,000	,000	,000	,000	,000	,000	,000	,000	,000	,000	,000	,000	,000	,000	,000	,000			
par_24	,000	,000	,000	,000	,001	,000	,000	,000	-,013	-,008	,000	,000	,000	-,004	-,002	-,001	-,001	,000	,000	,000	,000	,000	,000	,024		
par_25	,000	,000	,000	,000	,000	,000	,000	,000	,000	,000	,000	,000	,000	,000	,000	,000	,000	,000	,000	,000	,000	,000	,000	,000	,000	
par_26	,000	,000	,000	,000	-,003	-,001	-,003	,001	,000	,000	,000	,000	,000	-,048	-,023	,001	,002	,000	,000	,000	,000	,000	,000	,001	,000	,015
par_27	,000	,000	,000	,000	,000	,000	,000	,000	,000	,000	,000	,000	,000	,000	,000	,000	,000	,000	,000	,000	,000	,000	,000	,000	,000	,000
par_28	,000	,000	,000	,000	,000	,000	,000	,000	,000	,000	,000	,000	,000	,000	,000	,000	,000	,000	,000	,000	,000	,000	,000	,000	,000	,000
par_29	,000	,000	,000	,000	,000	,000	,000	,000	,000	,000	,000	,000	,000	,000	,000	,000	,000	,000	,000	,000	,000	,000	,000	,000	,000	,000
par_30	,000	,000	,000	,000	,000	,000	,000	,000	,000	,000	,000	,000	,000	,000	,000	,000	,000	,000	,000	,000	,000	,000	,000	,000	,000	,000
par_31	,000	,000	,000	,000	,000	,000	,000	,000	,000	,000	,000	,000	,000	,000	,000	,000	,000	,000	,000	,000	,000	,000	,000	,000	,000	,000
par_32	,000	,000	,000	,000	,000	,000	,000	,000	,000	,000	,000	,000	,000	,000	,000	,000	,000	,000	,000	,000	,000	,000	,000	,000	,000	,000
par_33	,000	,000	,000	,000	,000	,000	,000	,000	,000	,000	,000	,000	,000	,000	,000	,000	,000	,000	,000	,000	,000	,000	,000	,000	,000	,000
par_34	,000	,000	,000	,000	,000	,000	,000	,000	,000	,000	,000	,000	,000	,000	,000	,000	,000	,000	,000	,000	,000	,000	,000	,000	,000	,000
par_35	,000	,000	,000	,000	-,036	-,017	-,020	,004	,000	,000	,000	,003	,004	,004	,002	,001	,001	,000	,000	,000	,000	,000	,000	,000	,000	,001
par_36	,000	,000	,000	,000	,000	,000	,000	,000	,000	,000	,000	,000	,000	,000	,000	,000	,000	,000	,000	,000	,000	,000	,000	,000	,000	,000
par_37	,000	,000	,000	,000	,000	,000	,000	,000	,000	,000	,000	,000	,000	,000	,000	,000	,000	,000	,000	,000	,000	,000	,000	,000	,000	,000
par_38	,000	,000	,000	,000	,010	,005	,005	-,001	,000	,000	,000	-,001	-,001	-,003	-,001	-,001	,000	,000	,000	,000	,000	,000	,000	,000	,000	,000
par_39	,000	,000	,000	,000	-,101	,012	-,006	,005	,006	-,008	-,001	-,002	-,003	,012	-,010	,064	,073	,000	,000	,000	,000	,000	,000	-,005	,000	,002
par_40	,000	,000	,000	,000	,008	-,009	,002	,001	-,004	,001	,000	,001	,002	,006	,007	-,011	-,015	,000	,000	,000	,000	,000	,000	,001	,000	-,001
par_41	,000	,000	,000	,000	,002	,007	-,015	-,001	,006	,003	,000	-,001	-,001	-,015	-,010	,002	,004	,000	,000	,000	,000	,000	,000	-,001	,000	,003

Correlations of Estimates (Default model)

	par_1	par_2	par_3	par_4	par_5	par_6	par_7	par_8	par_9	par_10	par_11	par_12	par_13	par_14	par_15	par_16	par_17	par_18	par_19	par_20	par_21	par_22	par_23	par_24	par_25	par_26
par_1	1,000																									
par_2	,820	1,000																								
par_3	,751	,732	1,000																							
par_4	,625	,622	,560	1,000																						
par_5	,001	,000	,000	,000	1,000																					

par_6	-,001	,000	,000	,000	,831	1,000																				
par_7	,000	,000	,000	,000	,808	,829	1,000																			
par_8	,247	,246	,218	,187	-,525	-,536	-,477	1,000																		
par_9	-,006	,004	,002	,002	-,005	,022	-,056	-,061	1,000																	
par_10	,005	-,006	,001	-,002	,011	-,005	-,046	-,043	-,578	1,000																
par_11	,000	,000	,000	,000	-,003	-,004	-,009	,000	-,014	-,107	1,000															
par_12	,001	,000	,001	,000	-,447	-,520	-,471	,278	-,001	,003	-,001	1,000														
par_13	-,001	,001	,001	,001	-,485	-,575	-,511	,289	-,019	,018	-,001	,122	1,000													
par_14	,000	,000	,000	,000	-,075	-,076	-,004	,049	-,015	-,012	,002	,054	,066	1,000												
par_15	,000	,000	,000	,000	-,064	-,119	,000	,060	-,021	-,014	,004	,049	,057	,634	1,000											
par_16	-,001	,000	,000	,000	-,157	,024	-,013	,032	,005	-,009	,003	-,236	,043	-,076	-,012	1,000										
par_17	-,001	,000	,000	,001	-,162	,051	-,003	,023	,026	-,025	,003	,037	-,243	-,110	-,019	-,106	1,000									
par_18	,000	,000	,000	,000	,000	,000	,000	,000	,000	,000	,000	,000	,000	,000	,000	,000	,000	1,000								
par_19	,000	,000	,000	,000	,000	,000	,000	,000	,000	,000	,000	,000	,000	,000	,000	,000	,000	,290	1,000							
par_20	,000	,000	,000	,000	,000	,000	,000	,000	,000	,000	,000	,000	,000	,000	,000	,000	,000	,176	,212	1,000						
par_21	,000	,000	,000	,000	,000	,000	,000	,000	,000	,000	,000	,000	,000	,000	,000	,000	,000	,034	,033	,010	1,000					
par_22	,000	,000	,000	,000	,000	,000	,000	,000	,000	,000	,000	,000	,000	,000	,000	,000	,000	,004	,005	,025	,249	1,000				
par_23	,000	,000	,000	,000	,000	,000	,000	,000	,000	,000	,000	,000	,000	,000	,000	,000	,000	,005	,005	,027	,223	,315	1,000			
par_24	,002	,001	-,004	-,001	,019	,004	,014	-,030	-,364	-,221	,002	,003	,004	-,034	-,038	-,015	-,017	,000	,000	,000	,000	,000	,000	1,000		
par_25	-,219	-,200	-,189	-,166	,000	,000	,000	-,062	,001	-,001	-,006	-,001	,000	,000	,000	,000	,000	,000	,000	,000	,000	,000	,000	,000	1,000	
par_26	,001	,001	,001	,001	-,076	-,053	-,123	,059	,014	,009	-,001	-,040	-,065	-,574	-,649	,032	,043	,000	,000	,000	,000	,000	,000	,031	,000	1,000
par_27	,000	,000	,000	,000	,000	,000	,000	,000	,000	,000	,000	,000	,000	,000	,000	,000	,000	,037	,039	-,001	,001	,000	,000	,000	,000	,000
par_28	,000	,000	,000	,000	,000	,000	,000	,000	,000	,000	,000	,000	,000	,000	,000	,000	,000	,037	,011	,006	,393	,245	,070	,000	,000	,000
par_29	,000	,000	,000	,000	,000	,000	,000	,000	,000	,000	,000	,000	,000	,000	,000	,000	,000	,011	,039	,008	,393	,085	,293	,000	,000	,000
par_30	,000	,000	,000	,000	,000	,000	,000	,000	,000	,000	,000	,000	,000	,000	,000	,000	,000	,000	,000	-,001	,051	,245	,293	,000	,000	,000
par_31	-,832	-,810	-,731	-,615	,000	,000	,000	-,242	,000	,000	,001	,000	,000	,000	,000	,000	,000	,000	,000	,000	,000	,000	,000	,000	,195	-,001
par_32	-,194	,054	,004	,038	-,003	,003	,000	,010	,027	-,024	-,001	-,002	,004	,000	-,001	,003	,004	,000	,000	,000	,000	,000	,000	-,010	,030	,000
par_33	,047	-,167	-,007	-,027	,001	-,002	-,001	-,017	-,019	,024	,000	,003	-,003	,000	,000	-,002	-,002	,000	,000	,000	,000	,000	,000	-,001	-,024	,000
par_34	,007	,001	-,108	-,008	,001	,000	,001	,003	-,006	-,002	,000	-,001	-,002	,000	,000	,000	-,001	,000	,000	,000	,000	,000	,000	,011	,006	-,001
par_35	,001	-,001	,001	,000	-,885	-,915	-,866	,514	,007	,005	,005	,489	,535	,059	,076	,042	,029	,000	,000	,000	,000	,000	,000	-,008	,000	,076
par_36	,116	,113	,100	,070	,000	,000	,000	,032	,000	,000	,000	,000	,000	,000	,000	,000	,000	,000	,000	,000	,000	,000	,000	,000	-,020	,001
par_37	,033	,012	,012	-,074	,001	-,001	,000	,003	-,003	,002	-,001	,000	-,001	,000	,000	-,001	-,001	,000	,000	,000	,000	,000	,000	,001	,007	,000
par_38	,000	,000	,000	,000	,106	,113	,098	-,065	,002	-,001	-,002	-,056	-,070	-,016	-,019	-,007	,003	,000	,000	,000	,000	,000	,000	,004	,000	,000
par_39	-,002	,001	,000	,001	-,209	,056	-,020	,049	,021	-,026	-,005	-,021	-,033	,014	-,025	,152	,178	,000	,000	,000	,000	,000	,000	-,026	,001	,015
par_40	,003	-,001	,000	-,001	,095	-,233	,046	,035	-,073	,014	,002	,090	,126	,038	,101	-,153	-,201	,000	,000	,000	,000	,000	,000	,019	-,001	-,051
par_41	-,001	,000	,000	,000	,010	,066	-,122	-,021	,046	,024	,003	-,014	-,025	-,037	-,057	,009	,020	,000	,000	,000	,000	,000	,000	-,006	,000	,036

Estimates (Group number 1 - Default model)

Scalar Estimates (Group number 1 - Default model)

Maximum Likelihood Estimates

Regression Weights: (Group number 1 - Default model)

			Estimate	S.E.	C.R.	P	Label
total_PES	<---	BPSO	,166	,021	8,063	***	par_25
pes1particST	<---	total_PES	1,440	,046	31,264	***	par_1
pes2fundamST	<---	total_PES	1,229	,040	30,381	***	par_2
pes3habilST	<---	total_PES	1,382	,050	27,519	***	par_3
TOT_EBQ	<---	total_PES	,667	,073	9,153	***	par_8
TOT_EBQ	<---	FORM_PBE_150H	,541	,061	8,819	***	par_12
TOT_EBQ	<---	LEC_ART_MAYOR3	,570	,061	9,354	***	par_13
TOT_EBQ	<---	NivelEduc_Doc	,282	,121	2,327	,020	par_26
pes4dotaciST	<---	total_PES	1,000				
pes5relaciST	<---	total_PES	,971	,041	23,689	***	par_4
actitud	<---	TOT_EBQ	1,000				
conocimiento	<---	TOT_EBQ	5,440	,369	14,744	***	par_5
habilidades	<---	TOT_EBQ	2,612	,169	15,459	***	par_6
uso	<---	TOT_EBQ	3,169	,214	14,836	***	par_7
uso	<---	pes1particST	,944	,226	4,166	***	par_9
uso	<---	pes2fundamST	2,673	,240	11,119	***	par_10
uso	<---	BPSO	1,470	,206	7,120	***	par_11
conocimiento	<---	NivelEduc_Doc	4,556	,686	6,642	***	par_14
habilidades	<---	NivelEduc_Doc	,673	,289	2,325	,020	par_15
conocimiento	<---	FORM_PBE_150H	2,146	,322	6,656	***	par_16
conocimiento	<---	LEC_ART_MAYOR3	1,987	,313	6,351	***	par_17
uso	<---	pes3habilST	,815	,153	5,317	***	par_24

Standardized Regression Weights: (Group number 1 - Default model)

			Estimate
total_PES	<---	BPSO	,177
pes1particST	<---	total_PES	,896
pes2fundamST	<---	total_PES	,850
pes3habilST	<---	total_PES	,722
TOT_EBQ	<---	total_PES	,259
TOT_EBQ	<---	FORM_PBE_150H	,234
TOT_EBQ	<---	LEC_ART_MAYOR3	,262
TOT_EBQ	<---	NivelEduc_Doc	,069
pes4dotaciST	<---	total_PES	,595
pes5relaciST	<---	total_PES	,580
actitud	<---	TOT_EBQ	,353
conocimiento	<---	TOT_EBQ	,651
habilidades	<---	TOT_EBQ	,821
uso	<---	TOT_EBQ	,535
uso	<---	pes1particST	,099
uso	<---	pes2fundamST	,253
uso	<---	BPSO	,102
conocimiento	<---	NivelEduc_Doc	,133
habilidades	<---	NivelEduc_Doc	,052
conocimiento	<---	FORM_PBE_150H	,111
conocimiento	<---	LEC_ART_MAYOR3	,109
uso	<---	pes3habilST	,102

Covariances: (Group number 1 - Default model)

			Estimate	S.E.	C.R.	P	Label
BPSO	<-->	FORM_PBE_150H	,005	,004	1,264	,206	par_18
BPSO	<-->	LEC_ART_MAYOR3	,006	,004	1,348	,178	par_19
BPSO	<-->	NivelEduc_Doc	,000	,002	-,028	,978	par_20
FORM_PBE_150H	<-->	LEC_ART_MAYOR3	,066	,005	13,528	***	par_21
FORM_PBE_150H	<-->	NivelEduc_Doc	,021	,003	8,436	***	par_22
LEC_ART_MAYOR3	<-->	NivelEduc_Doc	,027	,003	10,085	***	par_23

Correlations: (Group number 1 - Default model)

			Estimate
BPSO	<-->	FORM_PBE_150H	,026
BPSO	<-->	LEC_ART_MAYOR3	,028
BPSO	<-->	NivelEduc_Doc	-,001
FORM_PBE_150H	<-->	LEC_ART_MAYOR3	,289
FORM_PBE_150H	<-->	NivelEduc_Doc	,176
LEC_ART_MAYOR3	<-->	NivelEduc_Doc	,212

Variances: (Group number 1 - Default model)

	Estimate	S.E.	C.R.	P	Label
BPSO	,196	,006	34,417	***	par_27
FORM_PBE_150H	,215	,006	34,417	***	par_28

LEC_ART_MAYOR3	,242	,007	34,417	***	par_29
NivelEduc_Doc	,069	,002	34,417	***	par_30
e6	,167	,011	15,191	***	par_31
e1	,088	,005	17,482	***	par_32
e2	,100	,004	22,871	***	par_33
e3	,303	,010	30,063	***	par_34
e11	,868	,109	7,937	***	par_35
e4	,316	,010	32,244	***	par_36
e5	,322	,010	32,330	***	par_37
e7	8,091	,243	33,280	***	par_38
e8	31,478	1,311	24,003	***	par_39
e9	3,598	,232	15,497	***	par_40
e10	16,623	,590	28,160	***	par_41

Squared Multiple Correlations: (Group number 1 - Default model)

	Estimate
total_PES	,031
TOT_EBQ	,245
pes3habilST	,521
pes2fundamST	,723
pes1particST	,803
uso	,589
habilidades	,691
conocimiento	,608
actitud	,124
pes5relaciST	,337
pes4dotaciST	,354

Matrices (Group number 1 - Default model)

Total Effects (Group number 1 - Default model)

	NivelEduc_Doc	LEC_ART_MAYOR3	FORM_PBE_150H	BPSO	total_PES	TOT_EBQ	pes3habilST	pes2fundamST	pes1particS
total_PES	,000	,000	,000	,166	,000	,000	,000	,000	,00
TOT_EBQ	,282	,570	,541	,111	,667	,000	,000	,000	,00
pes3habilST	,000	,000	,000	,229	1,382	,000	,000	,000	,00
pes2fundamST	,000	,000	,000	,204	1,229	,000	,000	,000	,00
pes1particST	,000	,000	,000	,239	1,440	,000	,000	,000	,00
uso	,893	1,806	1,716	2,779	7,885	3,169	,815	2,673	,94
habilidades	1,409	1,489	1,414	,289	1,743	2,612	,000	,000	,00
conocimiento	6,089	5,087	5,090	,603	3,629	5,440	,000	,000	,00
actitud	,282	,570	,541	,111	,667	1,000	,000	,000	,00
pes5relaciST	,000	,000	,000	,161	,971	,000	,000	,000	,00
pes4dotaciST	,000	,000	,000	,166	1,000	,000	,000	,000	,00

Standardized Total Effects (Group number 1 - Default model)

	NivelEduc_Doc	LEC_ART_MAYOR3	FORM_PBE_150H	BPSO	total_PES	TOT_EBQ	pes3habilST	pes2fundamST	pes1particS
total_PES	,000	,000	,000	,177	,000	,000	,000	,000	,00
TOT_EBQ	,069	,262	,234	,046	,259	,000	,000	,000	,00
pes3habilST	,000	,000	,000	,128	,722	,000	,000	,000	,00
pes2fundamST	,000	,000	,000	,150	,850	,000	,000	,000	,00
pes1particST	,000	,000	,000	,158	,896	,000	,000	,000	,00
uso	,037	,140	,125	,194	,516	,535	,102	,253	,09
habilidades	,108	,215	,192	,038	,212	,821	,000	,000	,00
conocimiento	,178	,279	,264	,030	,168	,651	,000	,000	,00
actitud	,024	,092	,083	,016	,091	,353	,000	,000	,00
pes5relaciST	,000	,000	,000	,103	,580	,000	,000	,000	,00
pes4dotaciST	,000	,000	,000	,105	,595	,000	,000	,000	,00

Direct Effects (Group number 1 - Default model)

	NivelEduc_Doc	LEC_ART_MAYOR3	FORM_PBE_150H	BPSO	total_PES	TOT_EBQ	pes3habilST	pes2fundamST	pes1particS
total_PES	,000	,000	,000	,166	,000	,000	,000	,000	,00
TOT_EBQ	,282	,570	,541	,000	,667	,000	,000	,000	,00
pes3habilST	,000	,000	,000	,000	1,382	,000	,000	,000	,00
pes2fundamST	,000	,000	,000	,000	1,229	,000	,000	,000	,00
pes1particST	,000	,000	,000	,000	1,440	,000	,000	,000	,00
uso	,000	,000	,000	1,470	,000	3,169	,815	2,673	,94
habilidades	,673	,000	,000	,000	,000	2,612	,000	,000	,00
conocimiento	4,556	1,987	2,146	,000	,000	5,440	,000	,000	,00
actitud	,000	,000	,000	,000	,000	1,000	,000	,000	,00
pes5relaciST	,000	,000	,000	,000	,971	,000	,000	,000	,00
pes4dotaciST	,000	,000	,000	,000	1,000	,000	,000	,000	,00

Standardized Direct Effects (Group number 1 - Default model)

	NivelEduc_Doc	LEC_ART_MAYOR3	FORM_PBE_150H	BPSO	total_PES	TOT_EBQ	pes3habilST	pes2fundamST	pes1particS
total_PES	,000	,000	,000	,177	,000	,000	,000	,000	,00
TOT_EBQ	,069	,262	,234	,000	,259	,000	,000	,000	,00
pes3habilST	,000	,000	,000	,000	,722	,000	,000	,000	,00
pes2fundamST	,000	,000	,000	,000	,850	,000	,000	,000	,00

pes1particST	,000	,000	,000	,000	,896	,000	,000	,000	,00
uso	,000	,000	,000	,102	,000	,535	,102	,253	,09
habilidades	,052	,000	,000	,000	,000	,821	,000	,000	,00
conocimiento	,133	,109	,111	,000	,000	,651	,000	,000	,00
actitud	,000	,000	,000	,000	,000	,353	,000	,000	,00
pes5relaciST	,000	,000	,000	,000	,580	,000	,000	,000	,00
pes4dotaciST	,000	,000	,000	,000	,595	,000	,000	,000	,00

Indirect Effects (Group number 1 - Default model)

	NivelEduc_Doc	LEC_ART_MAYOR3	FORM_PBE_150H	BPSO	total_PES	TOT_EBQ	pes3habilST	pes2fundamST	pes1particS'
total_PES	,000	,000	,000	,000	,000	,000	,000	,000	,00
TOT_EBQ	,000	,000	,000	,111	,000	,000	,000	,000	,00
pes3habilST	,000	,000	,000	,229	,000	,000	,000	,000	,00
pes2fundamST	,000	,000	,000	,204	,000	,000	,000	,000	,00
pes1particST	,000	,000	,000	,239	,000	,000	,000	,000	,00
uso	,893	1,806	1,716	1,309	7,885	,000	,000	,000	,00
habilidades	,736	1,489	1,414	,289	1,743	,000	,000	,000	,00
conocimiento	1,533	3,100	2,945	,603	3,629	,000	,000	,000	,00
actitud	,282	,570	,541	,111	,667	,000	,000	,000	,00
pes5relaciST	,000	,000	,000	,161	,000	,000	,000	,000	,00
pes4dotaciST	,000	,000	,000	,166	,000	,000	,000	,000	,00

Standardized Indirect Effects (Group number 1 - Default model)

	NivelEduc_Doc	LEC_ART_MAYOR3	FORM_PBE_150H	BPSO	total_PES	TOT_EBQ	pes3habilST	pes2fundamST	pes1particS'
total_PES	,000	,000	,000	,000	,000	,000	,000	,000	,00
TOT_EBQ	,000	,000	,000	,046	,000	,000	,000	,000	,00
pes3habilST	,000	,000	,000	,128	,000	,000	,000	,000	,00
pes2fundamST	,000	,000	,000	,150	,000	,000	,000	,000	,00
pes1particST	,000	,000	,000	,158	,000	,000	,000	,000	,00
uso	,037	,140	,125	,091	,516	,000	,000	,000	,00
habilidades	,057	,215	,192	,038	,212	,000	,000	,000	,00
conocimiento	,045	,170	,152	,030	,168	,000	,000	,000	,00
actitud	,024	,092	,083	,016	,091	,000	,000	,000	,00
pes5relaciST	,000	,000	,000	,103	,000	,000	,000	,000	,00
pes4dotaciST	,000	,000	,000	,105	,000	,000	,000	,000	,00
