# Supplementary material for: Determinants of Evidence Implementation by Nurses: #Evidencer Model for the Use of Evidence-Based Practice (#EvidencerMUSEBP)—A Structural Equation Model
Source: J Nurs Manag. 2024 Feb 28;2024:7246547. doi: 10.1155/2024/7246547 (PMC11919092; doi:10.1155/2024/7246547)
Supplement: Supplementary Materials — Estimates, covariance matrix, and correlation matrix of the study's initial and final models. [file 7246547.f1.zip › Initial model- Estimates - covariance matrix -correlation matrix (1).pdf]

Pairwise Parameter Comparisons (Default model)

Variance-covariance Matrix of Estimates (Default model)

	par_1	par_2	par_3	par_4	par_5	par_6	par_7	par_8	par_9	par_10	par_11	par_12	par_13	par_14	par_15	par_16	par_17	par_18	par_19	par_20	par_21	par_22	par_23	par_24	par_25	par_26
par_1	,002																									
par_2	,002	,002																								
par_3	,002	,001	,003																							
par_4	,001	,001	,001	,002																						
par_5	,000	,000	,000	,000	,199																					
par_6	,000	,000	,000	,000	,068	,028																				
par_7	,000	,000	,000	,000	,105	,040	,077																			
par_8	,001	,001	,001	,001	-,026	-,010	-,013	,007																		
par_9	,000	,000	,000	,000	-,016	-,006	-,010	,002	,003																	
par_10	,000	,000	,000	,000	-,016	-,006	-,010	,002	,001	,003																
par_11	,000	,000	,000	,000	,000	,000	,000	,000	,000	,000	,000															
par_12	,000	,000	,000	,000	-,015	-,006	-,010	,002	,001	,001	,000	,008														
par_13	,000	,000	,000	,000	,000	,000	,000	,000	,000	,000	,000	,000	,000													
par_14	,000	,000	,000	,000	,000	,000	,000	,000	,000	,000	,000	,000	,000	,000												
par_15	,000	,000	,000	,000	,000	,000	,000	,000	,000	,000	,000	,000	,000	,000	,000											
par_16	,000	,000	,000	,000	,000	,000	,000	,000	,000	,000	,000	,000	,000	,000	,000	,000										
par_17	,000	,000	,000	,000	,000	,000	,000	,000	,000	,000	,000	,000	,000	,000	,000	,000	,000									
par_18	,000	,000	,000	,000	,000	,000	,000	,000	,000	,000	,000	,000	,000	,000	,000	,000	,000	,000								
par_19	,000	,000	,000	,000	,002	,001	,001	,000	,000	,000	,000	,000	,000	,000	,000	,000	,000	,000	,003							
par_20	,000	,000	,000	,000	,000	,000	,000	,000	,000	,000	,000	,000	,000	,000	,000	,000	,000	,000	,000	,000						
par_21	,000	,000	,000	,000	,000	,000	,000	,000	,000	,000	,000	,000	,000	,000	,000	,000	,000	,000	,000	,000	,000					
par_22	,000	,000	,000	,000	,000	,000	,000	,000	,000	,000	,000	,000	,000	,000	,000	,000	,000	,000	,000	,000	,000	,000				
par_23	,000	,000	,000	,000	,000	,000	,000	,000	,000	,000	,000	,000	,000	,000	,000	,000	,000	,000	,000	,000	,000	,000	,000			
par_24	,000	,000	,000	,000	,000	,000	,000	,000	,000	,000	,000	,000	,000	,000	,000	,000	,000	,000	,000	,000	,000	,000	,000	,000		
par_25	,000	,000	,000	,000	,000	,000	,000	,000	,000	,000	,000	,000	,000	,000	,000	,000	,000	,000	,000	,000	,000	,000	,000	,000	,007	
par_26	,000	,000	,000	,000	,000	,000	,000	,000	,000	,000	,000	,000	,000	,000	,000	,000	,000	,000	,000	,000	,000	,000	,000	,000	,000	,008
par_27	,000	,000	,000	,000	,000	,000	,000	,000	,000	,000	,000	,000	,000	,000	,000	,000	,000	,000	,000	,000	,000	,000	,000	,000	-,001	,000
par_28	,000	,000	,000	,000	,000	,000	,000	,000	,000	,000	,000	,000	,000	,000	,000	,000	,000	,000	,000	,000	,000	,000	,000	,000	-,001	,000
par_29	,000	,000	,000	,000	,000	,000	,000	,000	,000	,000	,000	,000	,000	,000	,000	,000	,000	,000	,000	,000	,000	,000	,000	,000	,000	,000
par_30	,000	,000	,000	,000	,000	,000	,000	,000	,000	,000	,000	,000	,000	,000	,000	,000	,000	,000	,000	,000	,000	,000	,000	,000	,000	,000
par_31	,000	,000	,000	,000	,000	,000	,000	,000	,000	,000	,000	,000	,000	,000	,000	,000	,000	,000	,000	,000	,000	,000	,000	,000	,000	,000
par_32	,000	,000	,000	,000	,000	,000	,000	,000	,000	,000	,000	,000	,000	,000	,000	,000	,000	,000	,000	,000	,000	,000	,000	,000	,000	,000
par_33	,000	,000	,000	,000	,000	,000	,000	,000	,000	,000	,000	,000	,000	,000	,000	,000	,000	,000	,000	,000	,000	,000	,000	,000	,000	,000
par_34	,000	,000	,000	,000	,000	,000	,000	,000	,000	,000	,000	,000	,000	,000	,000	,000	,000	,000	,000	,000	,000	,000	,000	,000	,000	,000
par_35	,000	,000	,000	,000	,000	,000	,000	,000	,000	,000	,000	,000	,000	,000	,000	,000	,000	,000	,000	,000	,000	,000	,000	,000	-,014	,036
par_36	,000	,000	,000	,000	,000	,000	,000	,000	,000	,000	,000	,000	,000	,000	,000	,000	,000	,000	,000	,000	,000	,000	,000	,000	,000	,000
par_37	,000	,000	,000	,000	-,034	-,013	-,021	,004	,003	,003	,000	,003	,000	,000	,000	,000	,000	,000	,000	,000	,000	,000	,000	,000	,000	,000
par_38	,000	,000	,000	,000	,000	,000	,000	,000	,000	,000	,000	,000	,000	,000	,000	,000	,000	,000	,000	,000	,000	,000	,000	,000	,000	,000
par_39	,000	,000	,000	,000	,000	,000	,000	,000	,000	,000	,000	,000	,000	,000	,000	,000	,000	,000	,000	,000	,000	,000	,000	,000	,000	,000
par_40	,000	,000	,000	,000	,000	,000	,000	,000	,000	,000	,000	,000	,000	,000	,000	,000	,000	,000	,000	,000	,000	,000	,000	,000	,000	,000
par_41	,000	,000	,000	,000	,000	,000	,000	,000	,000	,000	,000	,000	,000	,000	,000	,000	,000	,000	,000	,000	,000	,000	,000	,000	,000	,000
par_42	,000	,000	,000	,000	,000	,000	,000	,000	,000	,000	,000	,000	,000	,000	,000	,000	,000	,000	,000	,000	,000	,000	,000	,000	,000	,000
par_43	,000	,000	,000	,000	,011	,004	,006	-,001	-,001	-,001	,000	-,001	,000	,000	,000	,000	,000	,000	,000	,000	,000	,000	,000	,000	,000	,000
par_44	,000	,000	,000	,000	-,130	-,007	,021	,027	-,002	-,001	,000	-,008	,000	,000	,000	,000	,000	,000	,002	,000	,000	,000	,000	,000	,000	,000
par_45	,000	,000	,000	,000	,003	-,004	,002	,001	,001	,001	,000	,001	,000	,000	,000	,000	,000	,000	,000	,000	,000	,000	,000	,000	,000	,000
par_46	,000	,000	,000	,000	,030	,004	-,031	-,014	,002	,001	,000	,004	,000	,000	,000	,000	,000	,000	-,001	,000	,000	,000	,000	,000	,000	,000

Correlations of Estimates (Default model)

	par_1	par_2	par_3	par_4	par_5	par_6	par_7	par_8	par_9	par_10	par_11	par_12	par_13	par_14	par_15	par_16	par_17	par_18	par_19	par_20	par_21	par_22	par_23	par_24	par_25	par_26

par_1	1,000																									
par_2	,822	1,000																								
par_3	,752	,733	1,000																							
par_4	,626	,623	,561	1,000																						
par_5	,001	-,001	-,001	-,001	1,000																					
par_6	-,001	,000	,000	,000	,901	1,000																				
par_7	,000	,003	,001	,001	,846	,865	1,000																			
par_8	,302	,304	,268	,227	-,704	-,683	-,575	1,000																		
par_9	,001	-,001	,001	,000	-,601	-,626	-,604	,427	1,000																	
par_10	-,001	,001	,001	,001	-,622	-,646	-,619	,428	,277	1,000																
par_11	-,222	-,204	-,192	-,168	-,001	,000	,002	-,073	-,001	-,001	1,000															
par_12	,003	,001	,002	,001	-,393	-,420	-,418	,302	,200	,176	-,001	1,000														
par_13	,000	,000	,000	,000	,000	,000	,000	,000	,000	,000	,000	,000	1,000													
par_14	,000	,000	,000	,000	,000	,000	,000	,000	,000	,000	,000	,000	,290	1,000												
par_15	,000	,000	,000	,000	,000	,000	,000	,000	,000	,000	,000	,000	,176	,212	1,000											
par_16	,000	,000	,000	,000	,000	,000	,000	,000	,000	,000	,000	,000	,005	,005	,027	1,000										
par_17	,000	,000	,000	,000	,000	,000	,000	,000	,000	,000	,000	,000	,034	,033	,010	,223	1,000									
par_18	,000	,000	,000	,000	,000	,000	,000	,000	,000	,000	,000	,000	,004	,005	,025	,315	,249	1,000								
par_19	,005	-,005	-,001	-,001	,077	,078	,091	-,063	-,025	,021	-,002	,017	,000	,000	,000	,000	,000	,000	1,000							
par_20	,000	,000	,000	,000	,000	,000	,000	,000	,000	,000	,000	,000	,007	,008	,038	-,151	-,040	-,101	,000	1,000						
par_21	,000	,000	,000	,000	,000	,000	,000	,000	,000	,000	,000	,000	,009	,034	,005	-,123	-,119	-,046	,000	,222	1,000					
par_22	,000	,000	,000	,000	,000	,000	,000	,000	,000	,000	,000	,000	,036	,008	,004	-,051	-,152	-,111	,000	,183	,297	1,000				
par_23	,000	,000	,000	,000	,000	,000	,000	,000	,000	,000	,000	,000	-,085	-,133	-,098	-,003	-,006	-,002	,000	-,004	,022	,023	1,000			
par_24	-,005	,000	,001	,005	,293	,318	,316	-,255	-,245	-,189	-,003	-,195	,000	,000	,000	,000	,000	,000	,065	,000	,000	,000	,000	1,000		
par_25	,000	,000	,000	,000	,000	,000	,000	,000	,000	,000	,000	,000	-,006	-,013	-,007	-,007	-,006	-,010	,000	,063	-,003	,054	,093	,000	1,000	
par_26	,000	,000	,000	,000	,000	,000	,000	,000	,000	,000	,000	,000	,053	-,005	,059	,002	,001	,001	,000	,002	-,001	,001	-,037	,000	,034	1,000
par_27	,000	,000	,000	,000	,000	,000	,000	,000	,000	,000	,000	,000	,096	,027	,018	,015	,006	,067	,000	-,012	-,011	-,045	-,009	,000	-,088	,031
par_28	,000	,000	,000	,000	,000	,000	,000	,000	,000	,000	,000	,000	,029	,095	,022	,056	,046	,027	,000	-,017	-,039	-,019	-,014	,000	-,134	,027
par_29	,000	,000	,000	,000	,000	,000	,000	,000	,000	,000	,000	,000	,017	,020	,095	,004	,009	,060	,000	-,046	-,008	-,012	-,009	,000	-,100	,005
par_30	,000	,000	,000	,000	,000	,000	,000	,000	,000	,000	,000	,000	,037	,011	,006	,070	,393	,245	,000	-,021	-,035	-,121	-,003	,000	-,006	,002
par_31	,000	,000	,000	,000	,000	,000	,000	,000	,000	,000	,000	,000	,011	,039	,008	,293	,393	,085	,000	-,040	-,188	-,055	-,005	,000	,002	,000
par_32	,000	,000	,000	,000	,000	,000	,000	,000	,000	,000	,000	,000	,037	,039	-,001	,000	,001	,000	,000	,000	,001	,001	,054	,000	,005	,134
par_33	,000	,000	,000	,000	,000	,000	,000	,000	,000	,000	,000	,000	,000	,000	-,001	,293	,051	,245	,000	-,138	-,029	-,024	,000	,000	-,008	,000
par_34	,000	,000	,000	,000	,000	,000	,000	,000	,000	,000	,000	,000	-,005	-,007	-,005	,018	,016	,012	,000	-,138	-,188	-,121	,054	,000	-,058	-,002
par_35	,000	,000	,000	,000	,000	,000	,000	,000	,000	,000	,000	,000	,007	-,001	,008	-,001	-,001	,004	,000	-,003	,000	-,003	-,005	,000	-,058	,134
par_36	-,832	-,812	-,731	-,615	,000	,000	-,001	-,298	,000	,000	,197	-,002	,000	,000	,000	,000	,000	,000	,001	,000	,000	,000	,000	,002	,000	,000
par_37	,001	-,002	,000	,000	-,911	-,903	-,887	,628	,600	,619	-,001	,403	,000	,000	,000	,000	,000	,000	-,082	,000	,000	,000	,000	-,302	,000	,000
par_38	-,185	,058	,004	,036	-,004	,001	,007	,017	-,005	,006	,031	-,005	,000	,000	,000	,000	,000	,000	-,022	,000	,000	,000	,000	,019	,000	,000
par_39	,044	-,168	-,007	-,026	,003	,000	-,008	-,027	,005	-,003	-,022	,004	,000	,000	,000	,000	,000	,000	,018	,000	,000	,000	,000	-,005	,000	,000
par_40	,005	,002	-,106	-,007	,001	,000	,000	,002	-,002	-,003	,007	-,002	,000	,000	,000	,000	,000	,000	,001	,000	,000	,000	,000	-,005	,000	,000
par_41	,113	,112	,098	,069	,000	,000	,001	,041	,000	,000	-,020	,002	,000	,000	,000	,000	,000	,000	,000	,000	,000	,000	,000	-,001	,000	,000
par_42	,031	,012	,012	-,072	,000	-,001	,000	,007	,001	-,001	,007	,001	,000	,000	,000	,000	,000	,000	,002	,000	,000	,000	,000	-,008	,000	,000
par_43	,000	,000	,000	,000	,104	,097	,092	-,073	-,061	-,069	,000	-,037	,000	,000	,000	,000	,000	,000	-,001	,000	,000	,000	,000	,039	,000	,000
par_44	-,003	,006	,002	,003	-,194	-,029	,051	,210	-,019	-,008	,004	-,058	,000	,000	,000	,000	,000	,000	,020	,000	,000	,000	,000	,050	,000	,000
par_45	,003	,003	,001	-,001	,036	-,119	,039	,069	,042	,047	,001	,030	,000	,000	,000	,000	,000	,000	,023	,000	,000	,000	,000	-,035	,000	,000
par_46	,001	-,007	-,003	-,002	,078	,029	-,132	-,188	,033	,025	-,005	,060	,000	,000	,000	,000	,000	,000	-,033	,000	,000	,000	,000	-,051	,000	,000

Estimates (Group number 1 - Default model)

Scalar Estimates (Group number 1 - Default model)

Maximum Likelihood Estimates

Regression Weights: (Group number 1 - Default model)

			Estimate	S.E.	C.R.	P	Label
total_PES	<---	BPSO	,168	,021	8,152	***	
TOT_EBQ	<---	total_PES	,939	,081	11,577	***	
TOT_EBQ	<---	FORM_PBE_150H	,614	,059	10,473	***	
TOT_EBQ	<---	LEC_ART_MAYOR3	,606	,057	10,682	***	
TOT_EBQ	<---	NivelEduc_Doc	,624	,087	7,190	***	
TOT_EBQ	<---	Sexo_Mujer	-,086	,050	-1,726	,084	
TOT_EBQ	<---	AñosFinEstudios	-,011	,002	-5,070	***	
pes1particST	<---	total_PES	1,441	,046	31,177	***	
pes2fundamST	<---	total_PES	1,234	,040	30,544	***	
pes3habilST	<---	total_PES	1,383	,050	27,553	***	
pes4dotaciST	<---	total_PES	1,000				
pes5relaciST	<---	total_PES	,971	,041	23,483	***	
actitud	<---	TOT_EBQ	1,000				
conocimiento	<---	TOT_EBQ	6,694	,440	15,212	***	
habilidades	<---	TOT_EBQ	2,565	,168	15,226	***	
uso	<---	TOT_EBQ	4,073	,276	14,754	***	

Standardized Regression Weights: (Group number 1 - Default model)

			Estimate
total_PES	<---	BPSO	,179
TOT_EBQ	<---	total_PES	,374
TOT_EBQ	<---	FORM_PBE_150H	,273
TOT_EBQ	<---	LEC_ART_MAYOR3	,286
TOT_EBQ	<---	NivelEduc_Doc	,157
TOT_EBQ	<---	Sexo_Mujer	-,033
TOT_EBQ	<---	AñosFinEstudios	-,101
pes1particST	<---	total_PES	,895
pes2fundamST	<---	total_PES	,853
pes3habilST	<---	total_PES	,722
pes4dotaciST	<---	total_PES	,594
pes5relaciST	<---	total_PES	,579
actitud	<---	TOT_EBQ	,343
conocimiento	<---	TOT_EBQ	,777
habilidades	<---	TOT_EBQ	,782
uso	<---	TOT_EBQ	,666

Covariances: (Group number 1 - Default model)

			Estimate	S.E.	C.R.	P	Label
FORM_PBE_150H	<-->	BPSO	,005	,004	1,264	,206	

LEC_ART_MAYOR3	<-->	BPSO	,006	,004	1,348	,178	
BPSO	<-->	NivelEduc_Doc	,000	,002	-,028	,978	
LEC_ART_MAYOR3	<-->	NivelEduc_Doc	,027	,003	10,085	***	
FORM_PBE_150H	<-->	LEC_ART_MAYOR3	,066	,005	13,528	***	
FORM_PBE_150H	<-->	NivelEduc_Doc	,021	,003	8,436	***	
NivelEduc_Doc	<-->	Sexo_Mujer	-,010	,002	-4,760	***	
LEC_ART_MAYOR3	<-->	Sexo_Mujer	-,026	,004	-6,471	***	
FORM_PBE_150H	<-->	Sexo_Mujer	-,016	,004	-4,169	***	
BPSO	<-->	Sexo_Mujer	,007	,004	1,856	,063	
Sexo_Mujer	<-->	AñosFinEstudios	-,165	,083	-1,990	,047	
BPSO	<-->	AñosFinEstudios	,422	,092	4,598	***	
FORM_PBE_150H	<-->	AñosFinEstudios	,236	,096	2,464	,014	
LEC_ART_MAYOR3	<-->	AñosFinEstudios	-,040	,101	-,390	,696	
NivelEduc_Doc	<-->	AñosFinEstudios	,156	,054	2,872	,004	

Correlations: (Group number 1 - Default model)

			Estimate
FORM_PBE_150H	<-->	BPSO	,026
LEC_ART_MAYOR3	<-->	BPSO	,028
BPSO	<-->	NivelEduc_Doc	-,001
LEC_ART_MAYOR3	<-->	NivelEduc_Doc	,212
FORM_PBE_150H	<-->	LEC_ART_MAYOR3	,289
FORM_PBE_150H	<-->	NivelEduc_Doc	,176
NivelEduc_Doc	<-->	Sexo_Mujer	-,098
LEC_ART_MAYOR3	<-->	Sexo_Mujer	-,134
FORM_PBE_150H	<-->	Sexo_Mujer	-,086
BPSO	<-->	Sexo_Mujer	,038
Sexo_Mujer	<-->	AñosFinEstudios	-,041
BPSO	<-->	AñosFinEstudios	,095
FORM_PBE_150H	<-->	AñosFinEstudios	,051
LEC_ART_MAYOR3	<-->	AñosFinEstudios	-,008
NivelEduc_Doc	<-->	AñosFinEstudios	,059

Variances: (Group number 1 - Default model)

	Estimate	S.E.	C.R.	P	Label
FORM_PBE_150H	,215	,006	34,417	***	
LEC_ART_MAYOR3	,242	,007	34,417	***	
BPSO	,196	,006	34,417	***	
NivelEduc_Doc	,069	,002	34,417	***	
Sexo_Mujer	,161	,005	34,417	***	
AñosFinEstudios	100,822	2,929	34,417	***	
e6	,167	,011	15,191	***	
e11	,636	,083	7,705	***	
e1	,089	,005	17,828	***	
e2	,099	,004	22,841	***	
e3	,303	,010	30,083	***	
e4	,316	,010	32,292	***	

e5	,322	,010	32,455	***
e7	8,162	,244	33,435	***
e8	31,914	1,422	22,438	***
e9	4,534	,206	22,061	***
e10	22,617	,798	28,338	***

Squared Multiple Correlations: (Group number 1 - Default model)

	Estimate
total_PES	,032
TOT_EBQ	,414
uso	,443
habilidades	,611
conocimiento	,604
actitud	,117
pes5relaciST	,335
pes4dotaciST	,353
pes3habilST	,521
pes2fundamST	,727
pes1particST	,801
